# A Risk Stratification Scheme for In-Hospital Cardiogenic Shock in Patients With Acute Myocardial Infarction

**DOI:** 10.3389/fcvm.2022.793497

**Published:** 2022-03-04

**Authors:** Jun-qing Yang, Peng Ran, Jie Li, Qi Zhong, Sidney C. Smith, Yan Wang, Gregg C. Fonarow, Jia Qiu, Louise Morgan, Xue-biao Wei, Xiao-bo Chen, Jie-leng Huang, Yong-chen Hao, Ying-ling Zhou, Chung-Wah Siu, Dong Zhao, Ji-yan Chen, Dan-qing Yu

**Affiliations:** ^1^Guangdong Provincial Key Laboratory of Coronary Heart Disease, Department of Cardiology, Guangdong Cardiovascular Institute, Guangdong Provincial People's Hospital, Guangdong Academy of Medical Sciences, Guangzhou, China; ^2^Division of Cardiology, University of North Carolina, Chapel Hill, NC, United States; ^3^Key Laboratory of Public Health Safety, School of Public Health, Fudan University, Ministry of Education, Shanghai, China; ^4^Division of Cardiology, Geffen School of Medicine at University of California, Los Angeles, Los Angeles, CA, United States; ^5^International Quality Improvement Department, American Heart Association, Dallas, TX, United States; ^6^Department of Pediatrics, Guangdong Provincial People's Hospital, Guangdong Academy of Medical Sciences, Guangzhou, China; ^7^Department of Epidemiology, Beijing Anzhen Hospital, Capital Medical University, Beijing Institute of Heart, Lung and Blood Vessel Diseases, Beijing, China; ^8^Cardiology Division, Department of Medicine, Queen Mary Hospital, The University of Hong Kong, Hong Kong, Hong Kong SAR, China

**Keywords:** acute myocardial infarction, cardiogenic shock, risk score, heart rate, heart failure

## Abstract

**Objective:**

Cardiogenic shock (CS) is the leading cause of death in patients with acute myocardial infarction (AMI) despite advances in care. This study aims to derive and validate a risk score for in-hospital development of CS in patients with AMI.

**Methods:**

In this study, we used the Improving Care for Cardiovascular Disease in China–Acute Coronary Syndrome (CCC–ACS) registry of 76,807 patients for model development and internal validation. These patients came from 158 tertiary hospitals and 82 secondary hospitals between 2014 and 2019, presenting AMI without CS upon admission. The eligible patients with AMI were randomly assigned to derivation (*n* = 53,790) and internal validation (*n* = 23,017) cohorts. Another cohort of 2,205 patients with AMI between 2014 and 2016 was used for external validation. Based on the identified predictors for in-hospital CS, a new point-based CS risk scheme, referred to as the CCC–ACS CS score, was developed and validated.

**Results:**

A total of 866 (1.1%) and 39 (1.8%) patients subsequently developed in-hospital CS in the CCC–ACS project and external validation cohort, respectively. The CCC–ACS CS score consists of seven variables, including age, acute heart failure upon admission, systolic blood pressure upon admission, heart rate, initial serum creatine kinase-MB level, estimated glomerular filtration rate, and mechanical complications. The area under the curve for in-hospital development of CS was 0.73, 0.71, and 0.85 in the derivation, internal validation and external validation cohorts, respectively.

**Conclusion:**

This newly developed CCC–ACS CS score can quantify the risk of in-hospital CS for patients with AMI, which may help in clinical decision making.

**Clinical Trial Registration:**

www.ClinicalTrials.gov, identifier: NCT02306616.

## Highlights

### What Is Already Known About This Subject?

- Cardiogenic shock (CS) is one of the leading causes of mortality in patients with acute myocardial infarction (AMI).
- The risk of CS in patients with AMI appears heterogeneous. Some risk scores, such as the Observatoire Régional Breton sur l'Infarctus (ORBI) risk score, were built to evaluate the risk of CS for selected patients with AMI.

### What Does This Study Add?

- Based on the data from a large published real-world cohort of patients with AMI, the new Improving Care for Cardiovascular Disease in China–Acute Coronary Syndrome (CCC–ACS) CS risk score was derived and validated with fewer variables compared with previous risk scores.
- The CCC–ACS CS risk score exhibited moderate discrimination and good calibration for predicting in-hospital CS across the entire spectrum of patients with AMI in the CCC–ACS cohort; it also performed well in an independent external AMI cohort.

### How Might This Have an Impact on Clinical Practice?

- The CCC–ACS CS risk score can quantify the risk of in-hospital CS for patients with AMI and may aid clinical decision-making, which may contribute to the efficient and appropriate allocation of medical resources.
- Distinguishing patients with AMI at different risk levels may help to screen candidates for future studies on preventing CS.

## Introduction

Cardiogenic shock (CS) is defined as systemic tissue hypoperfusion secondary to inadequate cardiac output despite the adequate circulatory volume and left ventricular filling pressure. Among patients with acute myocardial infarction (AMI), 5.5–13% develop CS, mostly during the index acute coronary syndrome (ACS) hospitalization (1–3), and it is the most common cause of death in patients with ACS (4, 5). The in-hospital mortality rate of patients who develop CS remains unacceptably high despite major advances, such as prompt revascularisation, in the management of AMI (1, 6–10). Furthermore, over the past decades, randomized controlled trials (RCTs) have failed to consistently demonstrate that percutaneous mechanical circulatory support improves survival (11–13). Therefore, strategies to prevent CS represent an important component of the management of AMI.

Conceivably, close monitoring in an intensive care unit (ICU) or a step-down ICU, together with prompt initiation of aggressive pharmacological and interventional therapies, may reduce CS, thereby improving the overall prognosis for patients with AMI. Nonetheless, indiscriminate use of intensive care can lead to overuse and waste of medical resources. Early and accurate identification of patients with AMI who are at high risk of CS will allow efficient and appropriate allocation of medical resources. However, the risk of CS in patients with AMI appears heterogeneous (14–16). To date, there are only a few existing risk stratification models for CS in patients with AMI, deriving primarily from RCT data with inclusion/exclusion criteria, or from registries of selected subsets of patients with AMI (14–16), which limits generalization ability to real-world AMI populations. The development of a risk stratification system for CS using a large and real-world AMI population with high discriminatory power may improve the decision-making process and streamline the management of patients with AMI to prevent CS. The Improving Care for Cardiovascular Disease in China–Acute Coronary Syndrome (CCC–ACS) project, an ongoing nationwide ACS registry, was initiated to prompt quality improvement for ACS care (17). We conducted this study to derive and validate a risk score for in-hospital development of CS in patients with AMI.

## Materials and Methods

### Study Design and Study Population

The data that support the findings of this study are available from the corresponding author upon reasonable request.

In this study, data from the CCC–ACS project were used as derivation and internal validation cohorts to build CCC–ACS in-hospital CS risk scores. The CCC–ACS project was developed by the American Heart Association (AHA) and the Chinese Society of Cardiology (CSC) and implemented by the Beijing Anzhen Hospital, Capital Medical University. The design and methodology of the project have been described previously (17), and the study was registered at www.clinicaltrials.gov (NCT02306616). The study protocol has been approved by the ethics committee of the Beijing Anzhen Hospital, Capital Medical University.

A total of 240 hospitals (158 tertiary hospitals and 82 secondary hospitals) from seven geographical regions of China (Northern, Northeast, Eastern, Central, Southern, Southwest and Northwest China) were recruited in phases I, II, III, and IV of the CCC–ACS project.

We used 76,807 patients with AMI from the CCC–ACS registry and randomly assigned them to derivation (*n* = 53,790) and internal validation (*n* = 23,017) cohorts. An independent external cohort of 2,205 patients with AMI between 2014 and 2016 was used for external validation.

Consecutive patients hospitalized with ACS in the participating hospitals during the study period were identified based on their principal diagnosis at discharge and retrospectively recruited to the CCC–ACS project. No informed consent was required. The investigation conforms to the principles outlined in the Declaration of Helsinki.

Patients hospitalized for ACS, including ST-segment elevation myocardial infarction (STEMI) and non-ST-segment elevation myocardial infarction (NSTEMI), between November 1, 2014 and December 31, 2019 who fulfilled the inclusion and exclusion criteria, were randomly assigned in a ratio of 7:3 to the derivation or internal validation cohort. Patients with AMI between May 1, 2014 and April 30, 2016 from the Guangdong Provincial People's Hospital (GDPH) were enrolled in an external validation cohort. GDPH is a tertiary hospital in southern China that participated in the CCC–ACS project. For the overlapping time periods, the first 20–30 consecutive patients with ACS each month were enrolled in the CCC–ACS project per protocol; subsequent patients were recruited for external validation.

Institutional review board approval was granted by the ethics committee of the GDPH. The Transparent Reporting of a multivariable prediction model for Individual Prognosis Or Diagnosis (TRIPOD) statement was followed for reporting multivariable prediction model derivation and validation (18).

### Inclusion and Exclusion Criteria

Inclusion criteria were as follows: (1) patients who were diagnosed with STEMI or NSTEMI; (2) age was older than 18 years old. Exclusion criteria were as follows: (1) patients who were diagnosed with unstable angina (UA); (2) patients with CS upon admission, or uncertain of onset time; (3) systolic blood pressure (SBP) upon admission was <90 mmHg.

### Data Collection

In the CCC–ACS project, demographics, medical history, clinical presentation, diagnosis, risk evaluation, in-hospital management, and outcomes of eligible patients were collected from medical charts by trained abstractors in participating hospitals and reported *via* a web-based platform (Oracle Clinical Remote Data Capture; Oracle Corporation, CA, USA). The reports were completed before the middle of the following month after corresponding patients were discharged. Quality audits by third-party clinical research associates were performed to ensure that cases were enrolled consecutively. Registry documents were compared with the original medical records in randomly selected cases (~5%) to ensure completeness and accuracy.

For the external validation cohort, the data were retrospectively collected from electronic medical records by trained medical staff; CS upon admission, in-hospital CS and in-hospital death were adjudicated by two experienced physicians.

### Diagnoses and Definitions

Cardiogenic shock refers to systemic tissue hypoperfusion secondary to inadequate cardiac output despite the adequate circulatory volume and left ventricular filling pressure. It is characterized haemodynamically according to the Chinese Guidelines for the Diagnosis and Treatment of Heart Failure 2014 (19). Because of the infrequent use of invasive haemodynamic measurements in real-world clinical practice, the diagnosis of CS was made by the individual attending physician and based on the following: (1) cardiac dysfunction assessed by the clinical symptoms, physical examination and laboratory examination; (2) SBP was <90 mmHg for over 30 min, necessitating inotrope and/or mechanical left ventricular support; (3) clinical features and/or laboratory evidence of tissue hypoperfusion, such as narrow pulse pressure, cold extremities, oliguria, disturbance of consciousness, metabolic acidosis or hyperlactataemia and/or correlated serum creatinine elevation.

The diagnoses and definitions of STEMI, NSTEMI, acute heart failure (AHF), mechanical complications (MCs), standardized value of initial creatine kinase-MB (CK-MB) level, and estimated glomerular filtration rate (eGFR) are described in the Supplementary Material.

### Laboratory Variables Measurement

The laboratory variables, including but not limited to serum creatinine (Scr), CK-MB and troponin T or I (TnT or I), were measured at local hospitals as routine practice, and the initial and peak values were collected in the CCC–ACS project.

### Study Outcome

The study outcome was the occurrence of CS after admission and throughout hospitalization. The study period started from the index date and ended with the occurrence of CS, death or hospital discharge, whichever came first.

### Statistical Analysis

The SAS version 9.4 software (SAS Institute, NC, USA) was used for statistical analysis. Normally distributed continuous data are presented as the mean ± SD, and non-normally distributed continuous data are presented as the median and interquartile range (IQR), which were compared using the *t*-test and the Mann–Whitney test, respectively. Categorical variables are displayed as numbers and percentages and were compared using the chi-square test.

The CCC–ACS CS risk score was built by fitting demographic, medical history, clinical and laboratory variables, which were selected based on their clinical significance and the findings from previous studies. Potential risk factors were screened through univariate logistic regression analysis with the level of significance set at *p* < 0.05. Multivariate stepwise logistic regression analysis was then performed to identify the best parsimonious set of these risk factors. For variables with a missing rate of <15% (e.g., Scr), except for CK-MB, we imputed missing values using the sequential regression multiple imputation method implemented by the IVEware software version 0.2 (Survey Research Center, University of Michigan, Ann Arbor, MI, USA) (Supplementary Material). Variables with a missing rate >15% were not imputed. The coefficient of individual variables was multiplied by a constant and rounded to the nearest integer, representing its weight. The risk score for in-hospital CS was then calculated for individual patients with AMI by adding the weights. The performance of the score for the prediction of in-hospital CS was further assessed using receiver operating characteristic (ROC) curves. Calibration was assessed by the Hosmer–Lemeshow goodness of fit test and calibration slope and intercept, with a slope of one and an intercept of zero indicating perfect calibration. All *p*-values were two tailed. A *p*-value of <0.05 was considered statistically significant.

### Patient and Public Involvement

On account of the retrospective nature of the CCC–ACS project, patients and the public have not been involved in the proposal of the research question, design, recruitment, and implementation of the study. The study results will be dispersed to the participants by public reporting.

## Results

### The General Characteristics

**Figure 1:**
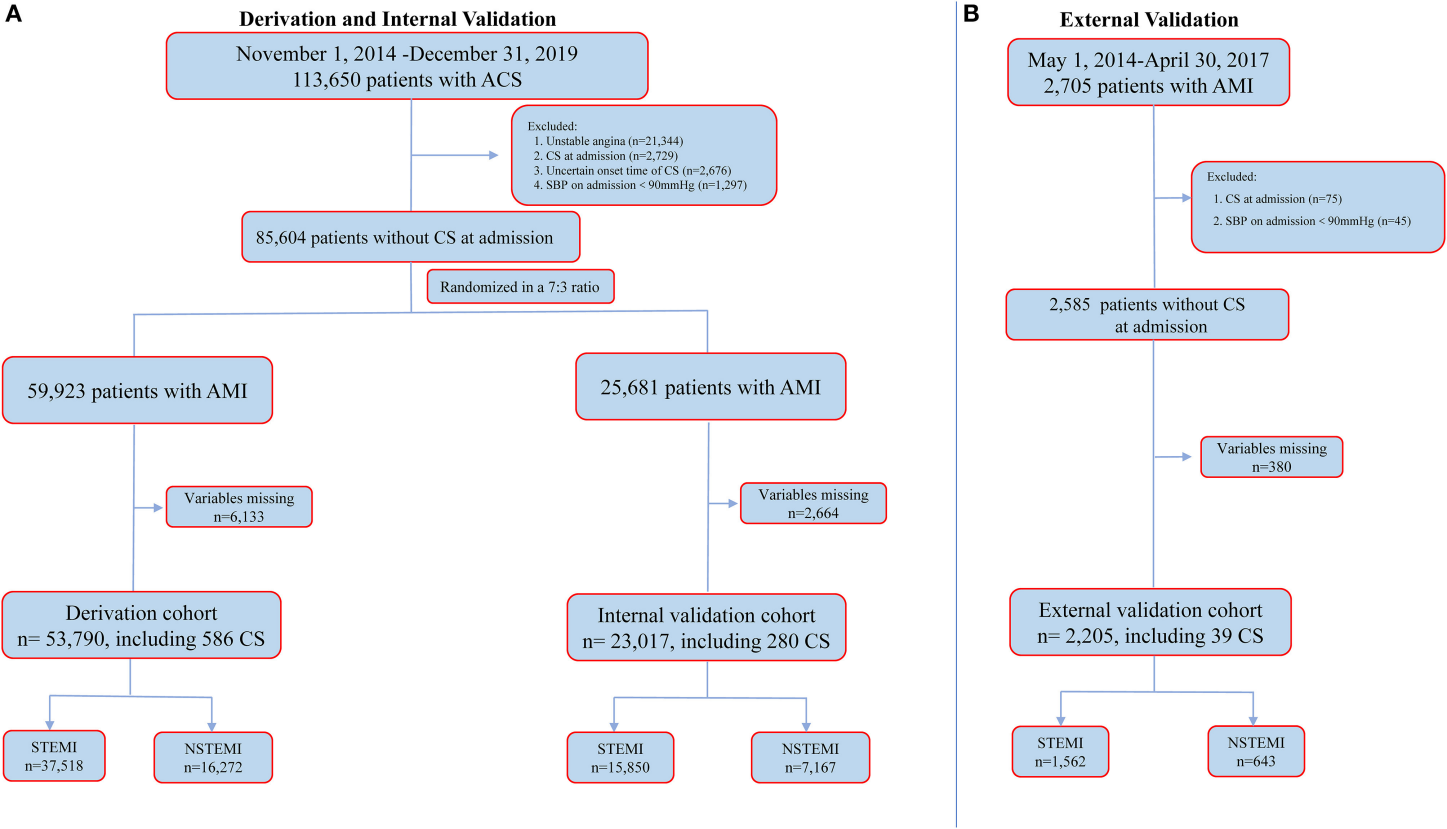
Cohort selection for the main analysis. **(A)** for CCC-ACS registry and **(B)** for external validation dataset. ACS, acute coronary syndrome; AMI, acute myocardial infarction; CS, cardiogenic shock; NSTEMI, non-ST-segment elevation myocardial infarction; SBP, systolic blood pressure; STEMI, ST-segment elevation myocardial infarction.

In the CCC–ACS project, a total of 113,650 patients who presented with ACS were enrolled. Of these patients, 21,344 patients with UA, 2,729 with CS upon admission, 2,676 with CS and uncertain onset time and 1,297 with a SBP <90 mmHg upon admission were excluded (Figure 1A). The resultant 85,604 patients with AMI were then randomly assigned in a 7:3 ratio to the derivation cohort or internal validation cohort. Of these patients, 6,133 (10.2%) from the derivation cohort and 2,664 (10.4%) from the internal validation cohort were excluded after randomization due to missing data pertinent to the final model.

Therefore, a total of 76,807 patients with AMI (age: 62.7 ± 12.6 years, male: 75.9%, STEMI: 69.5%) from the CCC–ACS project were included in the final analysis; 53,790 were in the derivation cohort and 23,017 were in the internal validation cohort (Figure 1A). A total of 866 (1.1%) patients developed in-hospital CS one (IQR 0–4 days) day after admission.

Table 1 summarizes the baseline characteristics, in-hospital management and outcomes in the derivation and internal validation cohorts. With the exception of types of AMI (*p* = 0.014), the derivation cohort and the validation cohort had similar in-patient characteristics, for which all standardized differences were >0.1.

**Table 1:** Patients clinical characteristics.

	**All(*n* = 76,807)**	**Derivation(*n* = 53,790)**	**Internal validation (*n* = 23,017)**	***P*-value^**§**^**
Age, years	62.7 ± 12.6	62.7 ± 12.6	62.7 ± 12.7	0.990
Female, %	18,518 (24.1%)	13,027 (24.2%)	5,491 (23.9%)	0.283
**Medical history**				
Prior myocardial infarction, %	5,544 (7.2%)	3,886 (7.2%)	1,658 (7.2%)	0.918
Prior CABG, %	316 (0.4%)	214 (0.4%)	102 (0.4%)	0.369
Prior PCI, %	4,931 (6.4%)	3,507 (6.5%)	1,424 (6.2%)	0.084
Hypertension, %	40,196 (52.3%)	28,121 (52.3%)	12,075 (52.5%)	0.644
Diabetes mellitus, %	16,917 (22.0%)	11,830 (22.0%)	5,087 (22.1%)	0.740
**Clinical conditions**				
Cardiac arrest, %	638 (0.8%)	460 (0.9%)	178 (0.8%)	0.252
AHF on admission, %	4,172 (5.4%)	2,912 (5.4%)	1,260 (5.5%)	0.734
Heart rate, beats/min	77.9 ± 15.9	77.9 ± 15.9	77.9 ± 15.8	0.831
Systolic blood pressure, mmHg	131.6 ± 22.6	131.6 ± 22.6	131.7 ± 22.6	0.657
Diastolic blood pressure, mmHg	79.3 ± 14.2	79.4 ± 14.2	79.3 ± 14.1	0.357
Time from symptom onset to admission, h^*^	8.6 (3.6, 39.0)	8.5 (3.6, 39.0)	8.7 (3.7, 39.4)	0.174
Time from symptom onset to admission^*^				0.852
<2 h, %	5,375 (10.2%)	3,795 (10.3%)	1,580 (10.1%)	
2–12 h, %	24,925 (47.4%)	17,523 (47.4%)	7,402 (47.4%)	
≥12 h, %	22,311 (42.4%)	15,665 (42.4%)	6,646 (42.5%)	
Time from admission to CS onset, days^*^	1 (0,4)	1 (0,4)	1 (0,4)	0.886
Mechanical complications, %	190 (0.2)	141 (0.3)	49 (0.2)	0.208
Types of AMI, %				0.014
STEMI	53,368 (69.5%)	37,518 (69.7%)	15,850 (68.9%)	
NSTEMI	23,439 (30.5%)	16,272 (30.3%)	7,167 (31.1%)	
**Laboratory variables**				
Serum creatinine, umol/L	75.9 (63.1, 92.0)	75.8 (63.0, 92.0)	76.0 (63.4, 92.0)	0.139
eGFR, ml/min/1.73 m^2^	92.5 ± 32.7	92.5 ± 32.6	92.3 ± 32.8	0.188
eGFR, ml/min/1.73 m^2^				0.277
≥30	74,749 (97.3%)	52,371 (97.4%)	22,378 (97.2%)	
<30 or prior dialysis	2,058 (2.7%)	1,419 (2.6%)	639 (2.8%)	
5 × elevated TnT or TnI, %^†^	45,429 (65.2%)	31,732 (65.1%)	13,697 (65.6%)	0.222
30 × elevated TnT or TnI, %^†^	29,328 (42.1%)	20,461 (42.0%)	8,867 (42.5%)	0.241
Initial CK-MB ≥10 × ULN, %	12,136 (15.8%)	8,441 (15.7%)	3,695 (16.1%)	0.209
ST-segment deviation, %	56,295 (73.3%)	39,369 (73.2%)	16,926 (73.5%)	0.320
LVEF^†^	55.0% ± 26.7%	55.1% ± 31.0%	54.8% ± 10.9%	0.064
**In-hospital therapy and outcomes**				
PCI, %	67,271 (87.6%)	47,118 (87.6%)	20,153 (87.6%)	0.880
Reperfusion therapy for STEMI, %	32,862 (61.6%)	23,058 (61.5%)	9,804 (61.9%)	0.653
Primary PCI, %	30,235 (56.7%)	21,235 (56.6%)	9,000 (56.8%)	
Fibrinolysis, %	2,017 (3.8%)	1,397 (3.7%)	620 (3.9%)	
Primary PCI+ Fibrinolysis, %	610 (1.1%)	426 (1.1%)	184 (1.2%)	
DTB within 90 min for STEMI^‡^, %	17,025 (66.4%)	11,965 (66.4%)	5,060 (66.4%)	0.990
Cardiogenic shock, %	866 (1.1%)	586 (1.1%)	280 (1.2%)	0.127
All-cause death, %	959 (1.2%)	663 (1.2%)	296 (1.3%)	0.541

*ACS, acute coronary syndromes; AHF, acute heart failure; CABG, coronary artery bypass grafting; CS, cardiogenic shock; DBP, diastolic blood pressure; DTB, door to balloon; eGFR, estimated glomerular filtration rate; LVEF, left ventricular ejection fraction; MI, myocardial infarction; NSTE-ACS, non-ST-segment elevation acute coronary syndromes; PCI, percutaneous coronary intervention; SBP, systolic blood pressure; STEMI, ST-segment elevation myocardial infarction; Tn, troponin*.

*Mechanical complications include free wall rupture, ventricular septal rupture, and papillary muscle-chordae rupture*.

* *Time from symptom onset to admission was not available for 1.4% (757/53,368) patients with STEMI in the total study population. Time from admission to CS onset was not available for 3.2% (28/866) patients with ACS in the study population*.

† *Investigation results were not available for TnT or TnI in 7,183 patients (9.4%) and LVEF in 14,470 patients (18.8%)*.

‡ *DTB time was not available for 16.8% (5,192/30,845) patients with STEMI who received primary PCI*.

§ *For continuous variables, student's t-test or the Mann–Whitney U-test were used to compare the clinical characteristics between two groups, while the Pearson χ^2^-test was used for categorical variables*.

Table 2 summarizes the clinical characteristics of patients in the derivation cohort with vs. without CS. Patients who developed in-hospital CS were older, more likely to be female, diabetic and had undergone coronary artery bypass graft (CABG). They were more likely to have STEMI as an index diagnosis, presented AHF upon admission, were in cardiac arrest and had MCs. They presented with lower blood pressure, faster heart rate, lower eGFR, lower left ventricular ejection fraction (LVEF) and higher CK-MB and troponin concentration; less were treated with percutaneous coronary intervention (PCI) or other reperfusion therapies (for STEMI) compared with those who did not develop CS. As expected, patients who developed in-hospital CS had a much higher in-hospital all-cause mortality rate (32.3 vs. 0.9%, *p* < 0.001).

**Table 2:** Clinical characteristics of patients in the derivation dataset with and without in-hospital cardiogenic shock.

	**In-hospital CS(*n* = 586)**	**No In-hospital CS(*n* = 53,204)**	***P*-value^**§**^**
Age, years	69.2 ± 11.8	62.6 ± 12.6	<0.001
Female, %	206 (35.2%)	12,821 (24.1%)	<0.001
**Medical history**			
Prior myocardial infarction, %	52 (8.9%)	3,834 (7.2%)	0.121
Prior CABG, %	7 (1.2%)	207 (0.4%)	0.009
Prior PCI, %	36 (6.1%)	3,471 (6.5%)	0.711
Hypertension, %	298 (50.9%)	27,823 (52.3%)	0.487
Diabetes mellitus, %	155 (26.5%)	11,675 (21.9%)	0.009
**Clinical conditions**			
Cardiac arrest, %	23 (3.9%)	437 (0.8%)	<0.001
Acute heart failure on admission, %	121 (20.6%)	2,912 (5.4%)	<0.001
Heart rate, beats/min	84.0 ± 20.7	77.8 ± 15.8	<0.001
Systolic blood pressure, mmHg	123.5 ± 23.2	131.7 ± 22.6	<0.001
Diastolic blood pressure, mmHg	75.5 ± 14.2	79.4 ± 14.2	<0.001
Time from symptom onset to admission^*^, h	10.2(4.0, 39.1)	8.5(3.6, 39.0)	0.129
Time from symptom onset to admission^*^			0.020
<2 h, %	30 (6.6%)	3,765 (10.3%)	
2–12 h, %	214 (47.0%)	17,309 (47.4%)	
≥12 h, %	211 (46.4%)	15,454 (42.3%)	
Mechanical complications, %	27 (4.6%)	114(0.2%)	<0.001
Types of AMI			<0.001
STEMI, %	460 (78.5%)	37,058 (69.7%)	
NSTE-ACS, %	126 (21.5%)	16,146 (30.3%)	
**Laboratory variables**			
Serum creatinine, umol/L	83.0 (68.0, 110.0)	75.6 (63.0, 92.0)	<0.001
eGFR, ml/min/1.73 m^2^	78.7 ± 34.1	92.7 ± 32.6	<0.001
eGFR, ml/min/1.73 m^2^			<0.001
≥30	537 (91.6%)	51,834 (97.4%)	
<30 or prior dialysis	49 (8.4%)	1,370 (2.6%)	
5 × elevated TnT or TnI^†^, %	356 (66.8%)	31,376 (65.1%)	0.411
30 × elevated TnT or TnI^†^, %	258 (48.4%)	20,203 (41.9%)	0.003
10 × elevated CK-MB, %	128 (21.8%)	8,313 (15.6)%	<0.001
ST-segment deviation	413 (70.5%)	38,956 (73.2%)	0.136
LVEF^†^	47.5% ± 12.2%	55.1% ± 31.2%	<0.001
**In-hospital therapy and outcomes**			
PCI, %	409 (69.8%)	46,709 (87.8%)	<0.001
Reperfusion therapy for STEMI, %	233 (50.7%)	22,825 (61.6%)	<0.001
Primary PCI, %	204 (44.3%)	21,031 (56.8%)	
Fibrinolysis, %	25 (5.4%)	1,372 (3.7%)	
Primary PCI+ Fibrinolysis, %	4 (0.9%)	422 (1.1%)	
DTB within 90 min for STEMI^‡^, %	121 (64.4%)	11,844 (66.4%)	0.558
All-cause death, %	189 (32.3%)	474 (0.9%)	<0.001

*ACS, acute coronary syndromes; AHF, acute heart failure; CABG, coronary artery bypass grafting; CS, cardiogenic shock; DBP, diastolic blood pressure; DTB, door to balloon; eGFR, estimated glomerular filtration rate; LVEF, left ventricular ejection fraction; MI, myocardial infarction; NSTE-ACS, non-ST-segment elevation acute coronary syndromes; PCI, percutaneous coronary intervention; SBP, systolic blood pressure; STEMI, ST-segment elevation myocardial infarction; Tn, troponin*.

* *Time from symptom onset to admission was not available for 1.4% (535/37,518) patients with STEM*.

† *Investigation results were not available for TnT or TnI in 5,050 patients (9.4%) and LVEF in 10,113 patients (18.8%)*.

‡ *DTB time was not available for 16.7% (3,633/21,661) patients with STEMI who received primary PCI*.

§ *For continuous variables, student's t-test or the Mann–Whitney U-test were used to compare the clinical characteristics between two groups, while the Pearson χ^2^-test was used for categorical variables*.

In the external validation cohort, a total of 2,705 patients with AMI were screened. Of these patients, 75 with CS upon admission, 45 with a SBP <90 mmHg upon admission and 380 with missing variables pertinent to the derived model were excluded. The resultant 2,205 patients with AMI (mean age: 61.7 ± 12.1, male: 83.1%, STEMI: 70.8%) were included in the final analysis (Figure 1B). A total of 39 (1.8%) patients developed in-hospital CS 2 (IQR 1–6) days after admission. More details are shown in Supplementary Table 1.

### Derivation and Validation of the CCC–ACS CS Risk Score

**Figure 2:**
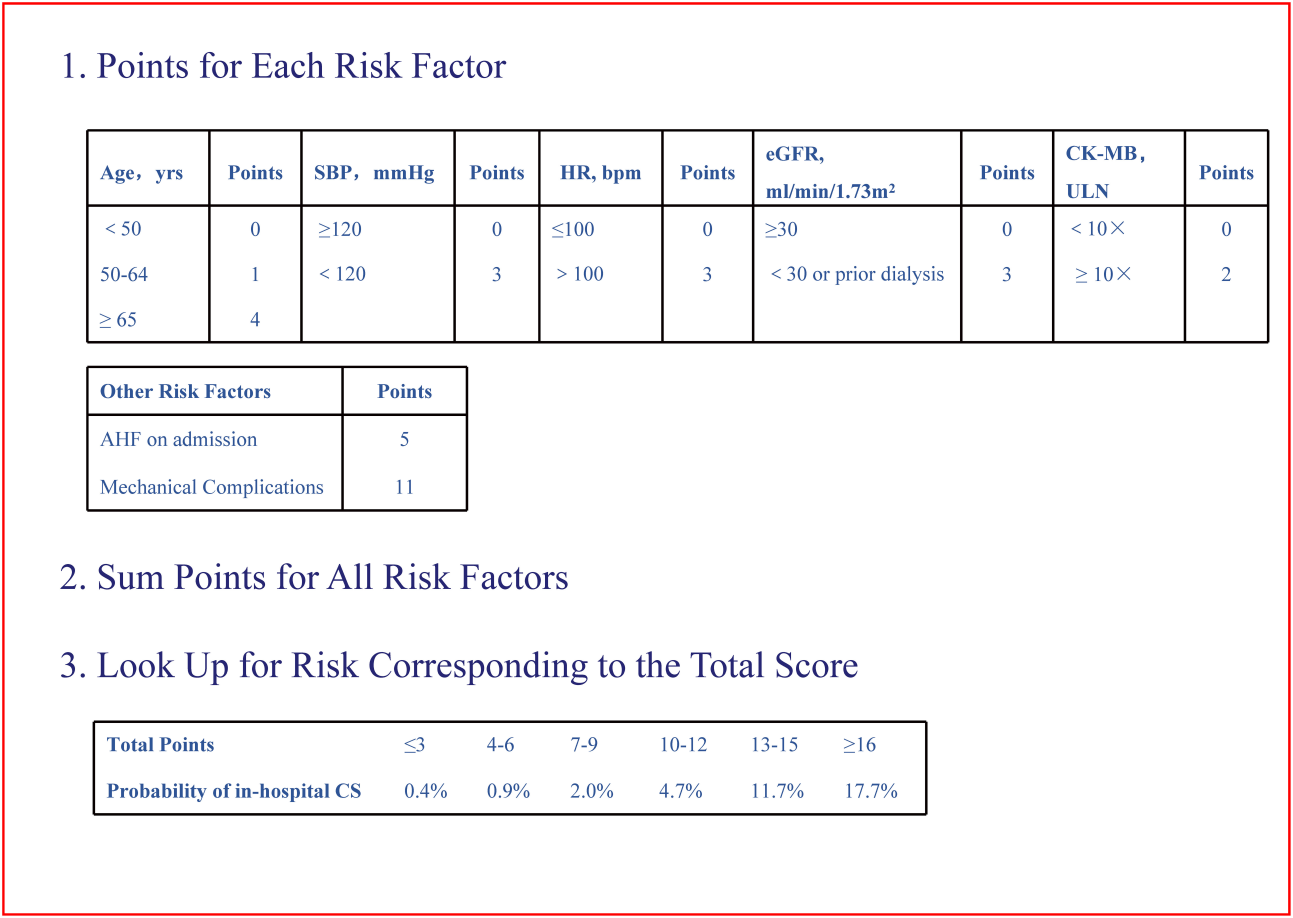
Improving care for cardiovascular disease in China–acute coronary syndrome cardiogenic shock risk score. CS, cardiogenic shock; CK-MB, creatine kinase-MB; eGFR, estimated glomerular filtration rate; HR, heart rate; SBP, systolic blood pressure; ULN, upper limit of normal.

**Figure 3:**
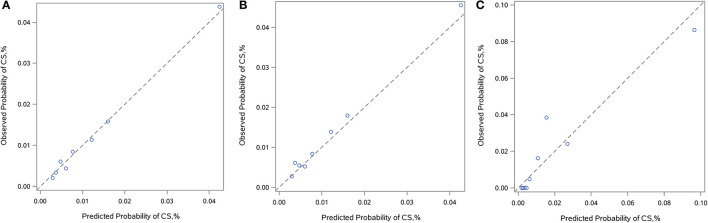
Calibration plots. Calibration plots showing observed vs. predicted incidence of in-hospital CS in the derivation **(A)**, internal **(B)**, and external **(C)** validation cohorts. The diagonal line indicates perfect calibration. CS, cardiogenic shock.

By logistic regression, the following were identified as independent predictors of in-hospital CS (Table 3 and Supplementary Table 2): age, SBP, heart rate, AHF upon admission, MCs, initial CK-MB level and eGFR. These factors were then used to construct a point-based risk scheme, referred to as the CCC–ACS CS score, to predict the development of in-hospital CS (Figure 2). The CCC–ACS CS score was the sum of the points for the presence of those predictors. Thus, the total score possibly ranged from 0 to 31. The area under the curve (AUC) of the original logistic model for predicting in-hospital CS was 0.73 and the χ^2^ statistic for calibration was 4.61 (*p* = 0.60). In the derivation cohort, the AUC of the CCC–ACS CS risk score was 0.73 and the χ^2^ statistic for calibration was 4.30 (*p* = 0.51). For the internal and external validation cohorts, the CCC–ACS CS score displayed moderate (AUC: 0.71) and good (AUC:0.85) discrimination, respectively. The CCC–ACS CS score displayed good validation by the Hosmer–Lemeshow goodness of fit test (internal validation: χ^2^ = 4.22, *p* = 0.52; external validation: χ^2^ = 8.13, *p* = 0.23); the slopes were 0.916 (*p* < 0.001) and 1.198 (*p* < 0.001), intercepts were −0.236 (*p* = 0.35), and 0.727 (*p* = 0.15) and the results (χ^2^ = 5.55, *p* = 0.06; χ^2^ = 2.14, *p* = 0.34) did not reject the null that the slope parameter is one and the intercept parameter is zero. Observation and prediction fit well in these cohorts (Figure 3).

**Table 3:** CCC–ACS cardiogenic shock risk score final model.

**Risk factors**	**β coefficient**	**χ^2^**	**OR**	**95% CI**	*P-*value^*^
**Age**					
<50 (reference)	–	–	–	–	–
50–64	0.36	4.11	1.43	1.01–2.02	0.0426
≥65	1.10	45.11	3.02	2.19–4.17	<0.0001
AHF on admission	1.15	107.21	3.16	2.54–3.93	<0.0001
SB*P < * 120 mmHg	0.74	75.68	2.10	1.78–2.48	<0.0001
Heart rate >100 bpm	0.81	55.07	2.26	1.82–2.80	<0.0001
Initial CK-MB ≥10 × ULN	0.44	18.18	1.55	1.27–1.89	<0.0001
eGFR <30 ml/min/1.73 m^2^ or Prior dialysis	0.84	28.22	2.31	1.70–3.15	<0.0001
Mechanical complications	2.64	130.96	13.94	8.88–21.90	<0.0001

*AHF, acute heart failure; CK-MB, creatine kinase-MB; eGFR, estimated glomerular filtration rate; SBP, systolic blood pressure; ULN, upper limit of normal*.

*Mechanical complications, including free wall rupture, ventricular septal rupture, and papillary muscle-chordae tendineae rupture*.

* *Multivariate stepwise logistic regression analysis was performed to identify risk factors for in-hospital cardiogenic shock with a p-value <0.05*.

### Risk Stratification and Model Performance in Subgroups

In the CCC–ACS registry, the proportion of patients who developed in-hospital CS increased progressively from 0.4% in patients with a score of 0–3 to 17.7% in those with a score ≥16 (*p* < 0.001).

Based on the observed rate of in-hospital CS stratified across different strata of the CCC–ACS CS scores, an individual patient's risk of in-hospital CS could be stratified accordingly: ≤6 low risk, 7–15 moderate risk, ≥16 high risk (Supplementary Figure 1). The proportion of patients were 79.2% (60,845) and 63.6% (1,402), 20.6% (15,798) and 35.0% (772), 0.2% (164), and 1.4% (31) in the CCC–ACS and external cohorts, respectively.

In addition, the CCC–ACS CS risk score also exhibited moderate discrimination among subgroups in the CCC–ACS cohort (Supplementary Table 3).

## Discussion

Based on the data of 85,604 real-world patients with AMI from the CCC–ACS project and 2,705 in an independent cohort, this study finds that at least 1.1–1.8% of patients developed in-hospital CS. We identified factors that predicted subsequent in-hospital CS amongst patients with AMI without CS upon admission. A new point-based CS risk scheme, referred to as the CCC–ACS CS score, was developed using readily available clinical parameters, including age, SBP, heart rate, eGFR, initial CK-MB level, AHF upon admission, and MCs. The in-hospital CS rate increased progressively with increasing CCC–ACS CS scores. The CCC–ACS CS score exhibited moderate and good predictive ability in the derivation and validation datasets across this large number of heterogeneous patients with AMI, with good calibration. Our data imply that the newly developed CCC–ACS CS score may be used to identify patients with AMI at high risk of in-hospital CS at an early stage, thereby guiding clinical management.

Several prior studies using data from large RCTs and registries have identified individual factors that predispose CS (15, 20–23). Concordantly, many of these factors, such as age, cardiovascular comorbidities, pre-existing cardiovascular disease, clinical and haemodynamic presentation, left ventricular systolic function and kidney function, were likewise found to be predictive of in-hospital CS in the present study. Although these individual factors are clearly associated with an increased relative risk of CS, or even of mechanistic importance to the development of CS, a comprehensive risk assessment to accurately quantify an individual patient's absolute risk of CS is the prerequisite to individualized management and prevention, particularly in the early stages.

The CCC–ACS CS model shared some risk factors with previous studies (14, 15, 21, 22, 24, 25) and also introduced some important factors, such as MCs. MCs are uncommon but can be life-threatening. They can cause acute haemodynamic changes leading to hypotension, AHF and CS (26). Therefore, without appropriate surgical intervention, MCs are commonly associated with early mortality. Elbadawi et al. demonstrated that MCs were strong risk factors for CS (27). However, MCs have not been incorporated into risk scores yet. As one of the strongest predictors in this study, MCs were incorporated into the CCC–ACS CS scores.

The effort has been made to derive a risk stratification scheme for CS in the presence of ACS (14, 15). Investigators from the Global Utilization of Streptokinase and Tissue Plasminogen Activator for Occluded Coronary Arteries (GUSTO-I) trial and the Global Use of Strategies to Open Occluded Coronary Arteries (GUSTO-III) trial that randomized patients with STEMI to different thrombolytic regimens developed a scoring system based primarily on the patient's age and physical examination findings on a presentation together with thrombolytic regimens that accurately quantified the absolute risk of in-hospital CS after thrombolytic therapy (15). Likewise, investigators from the Platelet Glycoprotein IIb/IIIa in UA: Receptor Suppression Using Integrilin Therapy (PURSUIT) trial that randomized patients with NSTE-ACS to eptifibatide or placebo have derived a scoring algorithm using similar clinical parameters to predict in-hospital CS (14). Nonetheless, patients from RCTs have typically been selected based on stringent inclusion and exclusion criteria and often are not comparable with the more heterogeneous patients of real-world clinical practice. For instance, the GUSTO trials included only patients with STEMI who presented within 6 h of the onset of chest pain and who were eligible for thrombolytic therapy, thereby limiting the generalization ability of results to much broader real-world STEMI populations, such as those with delayed presentation or with multiple comorbidities at the extremely high-risk end of the spectrum. Furthermore, current guidelines recommend PCI instead of thrombolytic therapy as the default mode of revascularisation for most patients with ACS. This has been widely adopted worldwide, further undermining the practical value of these scores in current clinical practice.

More recently, the Observatoire Régional Breton sur l'Infarctus (ORBI) risk score for in-hospital CS, which involves 11 variables, was shown to robustly stratify patients with STEMI treated by primary PCI for the risk of in-hospital development of CS (16). The ORBI's inclusion may potentially exclude patients with delayed presentation and/or poor premorbid states who are not suitable for primary PCI but who are at the highest risk of CS and mortality and who most require early risk stratification for appropriate triage. The integration of tissue perfusion and coronary anatomy parameters into the ORBI score makes early risk stratification only possible after coronary angiography and/or PCI, undermining the usefulness of the score. In contrast, in the CCC–ACS CS score, the all-comers design forming a large inclusive clinical registry for patients with AMI ensures a true representation of real-world patients with AMI. In addition, individual parameters constituting the score can be readily obtained during the initial clinical assessment, regardless of subsequent treatment. This allows easy clinical translation and rapid, widespread application in the real world. Certain components of the ORBI score are not available in the CCC–ACS and external validation cohorts; a direct comparison of their diagnostic performance is not possible. Despite fewer variables, the C-statistics for the CCC–ACS CS score (AUC 0.71–0.85) are at least comparable with those of the ORBI score (AUC 0.80).

### Strengths and Limitations

To the best of our knowledge, the CCC–ACS cohort represents the largest published real-world cohort of patients with AMI. The CCC–ACS CS score comprises seven simple clinical parameters readily available during the initial phase of AMI that allow early risk stratification, thereby potentially assisting decision-making management in the early stages. However, the study is limited by its registry-based observational design in primarily Chinese patients with AMI. The diagnosis of CS was made by the attending physician based on clinical judgment, without pre-specified haemodynamic criteria. Nonetheless, this is common practice in the real-world setting. Also despite these limitations, the incidence of CS and the associated mortality rate are consistent with those quoted in the current medical literature.

Moreover, despite the external validation in an independent AMI cohort, further validation in other populations remains necessary prior to the clinical adoption of the score since those data were obtained from Chinese patients. In addition, the ability to predict adverse outcomes in individual patients with AMI enables a more quantitative decision-making process that involves a delicate balance between potential benefits and adverse effects of therapeutic intervention, as well as resource allocation. Future clinical trials that incorporate this risk stratification scheme to guide clinical decision-making are the essential next step to ascertain its clinical usefulness. Finally, some parameters related to the PCI procedures [e.g., complete revascularisation, thrombolysis in myocardial infarction (TIMI) grade flow, etc.] or laboratory tests (e.g., NT-proBNP levels) were not collected and/or could not be incorporated into the CCC–ACS CS score, which may affect the discrimination power. However, established CCC–ACS CS scores displayed good discrimination ability and could be applied to a broader AMI population, such as those who do not undergo PCI.

## Conclusion

The CCC–ACS CS risk score consisting of seven readily available variables was derived and validated. The score may help to quantify the risk of in-hospital CS and stratify patients with AMI.

## Data Availability Statement

The original contributions presented in the study are included in the article/Supplementary Material, further inquiries can be directed to the corresponding author.

## Ethics Statement

The studies involving human participants were reviewed and approved by Guangdong Cardiovascular Institute, Guangdong Provincial People's Hospital.

## Author Contributions

J-qY, PR, and JL have made substantial contributions to conception and design. QZ, SS, YW, and GF did acquisition of data, analysis, and interpretation of data. J-qY, PR, JL, JQ, LM, X-bW, X-bC, and J-lH have been involved in drafting the manuscript and revising it critically for important intellectual content. Y-cH, Y-lZ, C-WS, DZ, J-yC, and D-qY have given final approval of the version to be published. All authors contributed to the article and approved the submitted version.

## Funding

The Improving Care for Cardiovascular Disease in China-Acute Coronary Syndrome (CCC–ACS) project is a collaborative program of the American Heart Association (AHA) and Chinese Society of Cardiology. The AHA was funded by Pfizer and Astra Zeneca for the quality improvement initiative through an independent grant for learning and change. This work was also supported by the Science and Technology Program of Guangzhou (No. 201704020124) and Sailing Foundation (Grant No. LHJJ201612127), Beijing, China. The funders had no role in study design, data collection and analysis, decision to publish, or preparation of the manuscript.

## Conflict of Interest

GF consulted for Abbott, Amgen, AstraZeneca, Bayer, Edwards, Janssen, Medtronic, Merck, and Novartis. The remaining authors declare that the research was conducted in the absence of any commercial or financial relationships that could be construed as a potential conflict of interest.

## Publisher's Note

All claims expressed in this article are solely those of the authors and do not necessarily represent those of their affiliated organizations, or those of the publisher, the editors and the reviewers. Any product that may be evaluated in this article, or claim that may be made by its manufacturer, is not guaranteed or endorsed by the publisher.

## Supplementary Material

Supplementary Figure 1 Observed incidence of in-hospital cardiogenic shock. Observed incidence of in-hospital cardiogenic shock according to categories of the Improving Care for Cardiovascular Disease in China–Acute Coronary Syndrome Cardiogenic Shock Risk Score in the CCC–ACS cohort and external validation cohort. Low (score ≤6), moderate (score 7–15), and high risk (score ≥16).

Supplementary Table 1 Clinical characteristics in external validation cohort.

Supplementary Table 2 Logistic regression for development of in-hospital CS.

Supplementary Table 3 Model performance in subgroups.

Supplementary Table 4 Investigators of CCC-ACS project.
