# Supplementary material for: A Risk Stratification Scheme for In-Hospital Cardiogenic Shock in Patients With Acute Myocardial Infarction
Source: Front Cardiovasc Med. 2022 Mar 4;9:793497. doi: 10.3389/fcvm.2022.793497 (PMC8931535; doi:10.3389/fcvm.2022.793497)
Supplement: Supplementary Table 1 — Clinical characteristics in external validation cohort. [file Data_Sheet_2.doc]

1. **Diagnoses and Definitions**
2. **Sensitivity Analysis**
3. **Some variables with a missing rate＜15% in the final analysis dataset**
4. **Supplementary Table S1. Clinical Characteristics in External Validation cohort.**
5. **Supplementary Table S2. Logistic regression for development of in-hospital CS.**
6. **Supplementary Table S3. Model Performance in Subgroups.**
7. **Supplementary Table S4. Investigators of CCC-ACS project.**

**1. The Diagnoses and Definitions**

**1) STEMI and NSTEMI**

The diagnosis of STEMI and NSTEMI are based on clinical symptoms, electrocardiogram (ECG) changes, and biomarker measurements as recommended by guideline for diagnosis and treatment of patients with ST-elevation myocardial infarction and guidelines for the diagnosis and treatment of non-ST-segment elevation acute coronary syndromes from the Chinese Society of Cardiology.

**2) Acute heart failure (AHF)**

The diagnosis of AHF was likewise made by the individual attending physician based on clinical symptoms and signs suggestive of congestion such as orthopnea, paroxysmal nocturnal dyspnea, bilateral pulmonary rales, bilateral peripheral edema, and elevated jugular venous pressure according to Chinese guidelines for the diagnosis and treatment of heart failure 2014.

**3) Mechanical complications (MCs)**

MCs was defined as free wall rupture, ventricular septal rupture, and/or papillary muscle-chordae tendineae rupture.

**4)** The obtained value of initial troponin level was divided by the upper limit of normal (ULN) for corresponding laboratory to gain a standardized value.

**5)** **Estimated glomerular filtration rate (eGFR):** the eGFR was calculated using the Modification of Diet in Renal Disease equation.

**2. Sensitivity Analysis**

Some of CS patients with incomplete information of onset time, who suffered from in-hospital CS actually, might bias the results. Therefore, all the group of 2,676 patients with CS but uncertain onset time were counted as in-hospital CS, added into the total analyzed CCC-ACS cohort, and then the sensitivity analysis was performed.

As the incidence of in-hospital CS increased to be 4.6% (3,542/79,483) in the CCC-ACS project. Therefore, the actual incidence of in-hospital CS was ranging from 1.1% to 4.6%, and AUC of the CCC-ACS CS risk score was 0.74.

1. **Some variables with a missing rate＜15% in the final analysis dataset**

Despite some variables with a missing rate＜15%, such as initial CK-MB level (10.3% , 8,797/85,604), time from symptom onset to admission (1.4% ,757/53,368), time from admission to CS onset (3.2%, 28/866) and TnT or TNI, these missing value were not imputed. There were several reasons: **First**, other variables were less associated with these missing data, and it was difficult to use existed variables to imput. **Second**, some variables (time from symptom onset to admission, time from admission to CS onset) had less impact for the model establishment. **Third**, some variables (initial CK-MB) were important to reflect the infarction size, severity of the illness and progression of diseases, and could be predicted by other variables. **Finally**, the misssing rate was ＞15% for some variables (TnT or TNI) in the CCC-ACS project.

1. **Supplementary Table S1. Clinical Characteristics in External Validation cohort**

**Supplementary Table S1. Clinical Characteristics in External Validation cohort**

| **Characteristic** | **N=2,205** |
| --- | --- |
| Age, years | 61.6±12.1 |
| Female, % | 376 (17.1%) |
| **Medical History** | |
| Prior myocardial infarction, % | 150 (6.8%) |
| Prior CABG, % | 14 (0.6%) |
| Prior PCI, % | 288 (13.1%) |
| Hypertension, % | 1,126 (51.1%) |
| Diabetes mellitus, % | 562 (25.5%) |
| **Clinical conditions** | |
| Cardiac arrest, % | 49 (2.2%) |
| AHF on admission, % | 532 (24.1%) |
| Heart rate, beats/min | 77±13 |
| Systolic blood pressure, mmHg | 126±19 |
| Diastolic blood pressure, mmHg | 75±12 |
| Time from symptom onset to admission, h* |  |
| Times from symptom onset to admission* |  |
| ＜2h, % | 24 (1.5%) |
| 2-12h, % | 221 (14.1%) |
| ≥12h, % | 1,317 (84.3%) |
| Times from admission to CS onset, days | 2 (1,6) |
| Mechanical complications, % | 17 (0.8%) |
| Types of AMI |  |
| STEMI, % | 1,562 (70.8%) |
| NSTEMI, % | 643 (29.2%) |
| **Laboratory variables** | |
| Scr, umol/L | 87.00 (74.10, 105.00) |
| eGFR, ml/min/1.73m^2^ | 80.44±30.89 |
| eGFR, ml/min/1.73m^2^ |  |
| ≥30 | 2,109 (95.6%) |
| ＜30 or prior dialysis | 96 (4.4%) |
| ≥5×elevated TnT or TnI † | 1,657 (77.9%) |
| ≥30×elevated TnT or TnI † | 1,008 (47.8%) |
| Initial CK-MB ≥10×ULN, % | 128 (5.8%) |
| ST-segment deviation, % | 1,645 (74.6%) |
| LVEF†, % | 54±11 |
| **In-hospital therapy** | |
| PCI, % | 1,900 (86.2%) |
| Reperfusion therapy for STEMI, % | 582 (37.3%) |
| Primary PCI, % | 331 (21.2%) |
| Fibrinolysis, % | 229 (14.7%) |
| Primary PCI+ Fibrinolysis, % | 22 (1.4%) |
| DTB within 90 min for STEMI§, % | -- |
| Cardiogenic shock, % | 39 (1.8%) |
| All-cause death, % | 44 (2.0%) |

**Abbreviations:** ACS, acute coronary syndromes. AHF, acute heart failure. CABG, coronary artery bypass grafting. CK-MB, creatine kinase-MB. CS, cardiogenic shock. DBP, diastolic blood pressure. DTB, door to balloon. eGFR, estimated glomerular filtration rate. LVEF, left ventricular ejection fraction. MI, Myocardial infarction. NSTE-ACS, non–ST-segment elevation acute coronary syndromes. PCI, percutaneous coronary intervention. SBP, systolic blood pressure. SCr, serum creatinine. STEMI, ST-segment elevation myocardial infarction. Tn, troponin.

Mechanical complications include free wall rupture, ventricular septal rupture, and papillary muscle-chordae rupture.

* Time from symptom onset to admission were not available for 0.4% (6/1,562) patients with STEMI in the external validation cohort.

† Investigation results were not available for TNI or TNT in 78 patients (3.5%) and LVEF in 387 patients (17.6%).

‡ DTB time for STEMI were not available for all patients in external validation cohort.

5. Supplementary Table S2. Logistic regression for development of in-hospital CS

| **Risk factors** | **Univariate analysis** | | |  | **Multivariate analysis** | | |
| --- | --- | --- | --- | --- | --- | --- | --- |
|  | **OR** | **95%CI** | ***P*** |  | **OR** | **95%CI** | ***P*** |
| **Age** |  |  |  |  |  |  |  |
| ＜50(reference) | -- | -- | -- |  | -- | -- | -- |
| 50-64 | 1.46 | 1.04-2.06 | 0.0307 |  | 1.42 | 1.01-2.01 | 0.0426 |
| ≥65 | 3.48 | 2.53-4.79 | <0.0001 |  | 2.99 | 2.17-4.13 | <0.0001 |
| **AHF on admission** | 4.70 | 3.83-5.76 | <0.0001 |  | 3.14 | 2.53-3.91 | <0.0001 |
| **SBP＜120 mmHg** | 2.07 | 1.76-2.44 | <0.0001 |  | 2.10 | 1.78-2.48 | <0.0001 |
| **Heart Rate＞100bpm** | 3.02 | 2.46-3.70 | <0.0001 |  | 2.26 | 1.82-2.80 | <0.0001 |
| **Initial CK-MB≥10×ULN** | 1.51 | 1.24-1.84 | <0.0001 |  | 1.55 | 1.27-1.90 | <0.0001 |
| **eGFR< 30 or Prior dialysis** | 3.45 | 2.57-4.65 | <0.0001 |  | 2.28 | 1.67-3.11 | <0.0001 |
| **Mechanical complications** | 22.50 | 14.67-34.51 | <0.0001 |  | 13.91 | 8.86-21.85 | <0.0001 |

**Abbreviation:** AHF: acute heart failure; CK-MB: creatine kinase-MB; eGFR: estimated glomerular filtration rate; SBP, systolic blood pressure; ULN: upper limit of normal.

Mechanical complications, including free wall rupture, ventricular septal rupture, and papillary muscle-chordae tendineae rupture.

**6.Supplementary Table S3.** **Model Performance in Subgroup**s

| **Subgroup** | **Sample size** | **AUC** |
| --- | --- | --- |
| **All** | 76,807 | 0.73 |
| **Gender** |  |  |
| Female | 18,518 (24.1%) | 0.72 |
| Male | 58,289 (75.9%) | 0.72 |
| **DM** |  |  |
| Yes | 16,917 (22.0%) | 0.76 |
| No | 59,890 (78.0%) | 0.71 |
| **Hypertension** |  |  |
| Yes | 40,196 (52.3%) | 0.73 |
| No | 36,611 (46.5%) | 0.72 |
| **AMI type** |  |  |
| STEMI | 53,368(69.5%) | 0.72 |
| NSTEMI | 23,439 (30.5%) | 0.73 |
| **LVEF missing** |  |  |
| Yes | 14,470 (18.8%) | 0.75 |
| No | 62,237 (81.2%) | 0.71 |
| **PCI** |  |  |
| Yes | 67,271(71.1%) | 0.71 |
| No | 26,204(28.9%) | 0.72 |
| **Primary PCI for STEMI** |  |  |
| Yes | 30845（57.8%） | 0.71 |
| No | 22523（42.2%） | 0.74 |

**Abbreviation:** ACS, acute coronary syndrome. AUC, area under the curve. DM, Diabetes Mellitus. MCs, mechanical complications. NSTEMI, non-ST-segment elevation myocardial infarction. PCI, percutaneous coronary intervention. STEMI, ST-segment elevation myocardial infarction. UA, unstable angina.

7.Supplementary Table S4. Investigators of CCC-ACS project

| **ID** | **Hospitals** | **Territories** | **Provinces** | **City** | **Investigator** |
| --- | --- | --- | --- | --- | --- |
| 1 | Shanxi Cardiovascular Hospital | Northern China | Shanxi | Taiyuan | Bao Li |
| 2 | Nanjing Drum Tower Hospital, The Affiliated Hospital of Nanjing University Medical School | Eastern China | Jiangsu | Nanjing | Biao Xu, Guangshu Han |
| 3 | Hainan General Hospital | Southern China | Hainan | Haikou | Bin Li |
| 4 | The Second Hospital of Jilin University | Northeast China | Jilin | Changchun | Bin Liu |
| 5 | The 2nd Affiliated Hospital of Harbin Medical University | Northeast China | Heilongjiang | Harbin | Bo Yu |
| 6 | The Ninth Hospital Affiliated to Shanghai Jiaotong University School of Medicine | Eastern China | Shanghai | Shanghai | Changqian Wang |
| 7 | Henan Provincial People’s Hospital | Central China | Henan | Zhengzhou | Chuanyu Gao |
| 8 | Shanxi Provincial People’s Hospital | Northern China | Shanxi | Taiyuan | Chunlin Lai |
| 9 | Xinqiao Hospital, Third Military Medical University | Southwest China | Chongqing | Chongqing | Cui Bin, Lan Huang |
| 10 | China Meitan General Hospital | Northern China | Beijing | Beijing | Di Wu |
| 11 | The 309th Hospital of Chinese People’s Liberation Army | Northern China | Beijing | Beijing | Fakuan Tang, Jun Xiao |
| 12 | Zhongda Hospital, Southeast University | Eastern China | Jiangsu | Nanjing | Genshan Ma |
| 13 | The First Affiliated Hospital of Liaoning Medical University | Northeast China | Liaoning | Jinzhou | Guizhou Tao |
| 14 | Xinjiang Uygur Autonomous Region People’s Hospital | Northwest China | Xinjiang | Urumchi | Guoqing Li |
| 15 | Sir Run Run Shaw Hospital, College of Medicine, Zhejiang University | Eastern China | Zhejiang | Hangzhou | Guosheng Fu |
| 16 | Beijing Friendship Hospital, Capital Medical University | Northern China | Beijing | Beijing | Hongwei Li |
| 17 | The First Affiliated Hospital of Bengbu Medical College | Eastern China | Anhui | Bengbu | Honhju Wang |
| 18 | General Hospital of TISCO | Northern China | Shanxi | Taiyuan | Huifeng Wang |
| 19 | Dongguan People’s Hospital | Southern China | Guangdong | Dongguan | Jianfeng Ye |
| 20 | Panyu Hospital of Chinese Medicine | Southern China | Guangdong | Guangzhou | Jianhao Li |
| 21 | Peking University First Hospital | Northern China | Beijing | Beijing | Jie Jiang |
| 22 | Sun Yat-sen Memorial Hospital, Sun Yat-sen University | Southern China | Guangdong | Guangzhou | Jingfeng Wang |
| 23 | Guangdong Provincial People's Hospital | Southern China | Guangdong | Guangzhou | Jiyan Chen |
| 24 | Hospital of Xinjiang Production & Construction Corps | Northwest China | Xinjiang | Urumchi | Junming Liu |
| 25 | The Military General Hospital of Beijing PLA | Northern China | Beijing | Beijing | Junxia Li |
| 26 | The First Affiliated Hospital of Guangxi Medical University | Southern China | Guangxi | Nanning | Lang Li |
| 27 | Tongren Hospital Affiliated to Shanghai Jiaotong University School of Medicine | Eastern China | Shanghai | Shanghai | Li Jiang |
| 28 | Binzou City Center Hospital | Eastern China | Shandong | Binzhou | Lijun Meng |
| 29 | The First Affiliated Hospital of Zhengzhou University | Central China | Henan | Zhengzhou | Ling Li |
| 30 | Xijing Hospital | Northwest China | Shaanxi | Xi’an | Ling Tao |
| 31 | The Affiliated Hospital of Guizhou Medical University | Southwest China | Guizhou | Guiyang | Lirong Wu |
| 32 | First Affiliated Hospital of the People’s Liberation Army General Hospital | Northern China | Beijing | Beijing | Miao Tian |
| 33 | The Second People’s Hospital of Yunnan Province | Southwest China | Yunnan | Kunming | Minghua Han |
| 34 | Haikou People’s Hospital | Southern China | Hainan | Haikou | Moshui Chen |
| 35 | Gansu Provincial Hospital | Northwest China | Gansu | Lanzhou | Ping Xie |
| 36 | The First Affiliated Hospital of Henan University of Science and Technology | Central China | Henan | Luoyang | Pingshuan Dong |
| 37 | Chenzhou First People’s Hospital | Central China | Hunan | Chenzhou | Qiaoqing Zhong |
| 38 | People’s Hospital of Qinghai Province | Northwest China | Qinghai | Xining | Rong Chang |
| 39 | Affiliated Hospital of Ningxia Medical University | Northwest China | Ningxia | Yinchuan | Shaobin Jia |
| 40 | Beijing Anzhen Hospital, Capital Medical University | Northern China | Beijing | Beijing | ShaopingNie, Xiaohui Liu |
| 41 | North Jiangsu People’s Hospital | Eastern China | Jiangsu | Yangzhou | Shenghu He |
| 42 | Shanghai Sixth People’s Hospital | Eastern China | Shanghai | Shanghai | Shixin Ma |
| 43 | The First Hospital of Handan | Northern China | Hebei | Handan | Shuanli Xin |
| 44 | Huai’an First People’s Hospital | Eastern China | Jiangsu | Huai’an | Shuren Ma |
| 45 | The First Affiliated Hospital of Chongqing Medical University | Southwest China | Chongqing | Chongqing | Suxin Luo |
| 46 | Navy General Hospital | Northern China | Beijing | Beijing | Tianchang Li |
| 47 | Zhejiang Provincial Hospital of TCM | Eastern China | Zhejiang | Hangzhou | Wei Mao |
| 48 | The Third Xiangya Hospital of Central South University | Central China | Hunan | Changsha | Weihong Jiang |
| 49 | Affiliated Hospital of Qinghai University | Northwest China | Qinghai | Xining | Weijun Liu |
| 50 | Teda International Cardiovascular Hospital | Northern China | Tianjin | Tianjin | Wenhua Lin |
| 51 | The Second Hospital of Hebei Medical University | Northern China | Hebei | Shijiazhuang | Xianghua Fu |
| 52 | Changhai Hospital of Shanghai | Eastern China | Shanghai | Shanghai | Xianxian Zhao |
| 53 | The Second Affiliated Hospital to Nanchang University | Eastern China | Jiangxi | Nanchang | Xiaoshu Cheng |
| 54 | Hebei General Hospital | Northern China | Hebei | Shijiazhuang | Xiaoyong Qi |
| 55 | Inner Mongolia People’s Hospital | Northern China | Inner Mongolia | Hohhot | Xingsheng Zhao |
| 56 | The General Hospital of Shenyang Military Region | Northeast China | Liaoning | Shenyang | Yaling Han |
| 57 | The First Hospital of Jilin University | Northeast China | Jilin | Changchun | Yang Zheng |
| 58 | Tianjin Chest Hospital | Northern China | Tianjin | Tianjin | Yin Liu |
| 59 | Hunan Provincial People’s Hospital | Central China | Hunan | Changsha | Ying Guo |
| 60 | People’s Hospital of Yuxi City | Southwest China | Yunnan | Yuxi | Yinglu Hao |
| 61 | The People’s Hospital of Guangxi Zhuang Autonomous Region | Southern China | Guangxi | Nanning | Yingzhong Lin |
| 62 | The First Teaching Hospital of Xinjiang Medical University | Northwest China | Xinjiang | Urumchi | Yitong Ma |
| 63 | Baogang Hospital | Northern China | Inner Mongolia | Baotou | Yongdong Li |
| 64 | Tianjin Medical University General Hospital | Northern China | Tianjin | Tianjin | Yuemin Sun |
| 65 | The Second Affiliated Hospital of Zhengzhou University | Central China | Henan | Zhengzhou | Yulan Zhao |
| 66 | Nanfang Hospital of Southern Medical University | Southern China | Guangdong | Guangzhou | Yuqing Hou |
| 67 | The First Affiliated Hospital to Nanchang University | Eastern China | Jiangxi | Nanchang | Zeqi Zheng |
| 68 | The First Affiliated Hospital of Lanzhou University | Northwest China | Gansu | Lanzhou | Zheng Zhang |
| 69 | The Third Hospital of Shijiazhuang | Northern China | Hebei | Shijiazhuang | Zhenguo Ji |
| 70 | Wuxi People’s Hospital | Eastern China | Jiangsu | Wuxi | Zhenyu Yang |
| 71 | Jiangsu Province Hospital | Eastern China | Jiangsu | Nanjing | Zhijian Yang |
| 72 | The Second Hospital of Shanxi Medical University | Northern China | Shanxi | Taiyuan | Zhiming Yang |
| 73 | The Affiliated Hospital of Xuzhou Medical College | Eastern China | Jiangsu | Xuzhou | Zhirong Wang |
| 74 | Southwest Hospital, Third Military Medical University | Southwest China | Chongqing | Chongqing | Zhiyuan Song |
| 75 | The First Affiliated Hospital of Xi’an Jiaotong University | Northwest China | Shaanxi | Xi’an | Zuyi Yuan |
| 76 | Yangzhou First People’s Hospital | Eastern China | Jiangsu | Yangzhou | Aihua Li |
| 77 | Hospital 463 of Chinese People’s Liberation Army | Northeast China | Liaoning | Shenyang | Bosong Yang |
| 78 | The Central Hospital of Mianyang | Northwest China | Sichuan | Mianyang | Caidong Luo |
| 79 | Liaocheng People’s Hospital | Eastern China | Shandong | Liaocheng | Chunyan Zhang |
| 80 | Yancheng Third People’s Hospital | Eastern China | Jiangsu | Yancheng | Chunyang Wu |
| 81 | The Second Xiangya Hospital of Central South University | Central China | Hunan | Changsha | Daoquan Peng |
| 82 | The Central Hospital of Panzhihua | Northwest China | Sichuan | Panzhihua | Dawen Xu |
| 83 | The First Hospital of Qiqihar City | Northeast China | Heilongjiang | Qiqihar | Gang Xu |
| 84 | The Third the People’s Hospital of Bengbu | Eastern China | Anhui | Bengbu | Gengsheng Sang |
| 85 | The First Hospital of Jiamusi | Northeast China | Heilongjiang | Jiamusi | Guixia Zhang |
| 86 | Zhoushan People’s Hospital | Eastern China | Zhejiang | Zhoushan | Guoxiong Chen |
| 87 | Dalian Municipal Central Hospital | Northeast China | Liaoning | Dalian | Hailong Lin |
| 88 | Renmin Hospital of Wuhan University | Central China | Hubei | Wuhan | Hong Jiang |
| 89 | Ningxia People’s Hospital | Northwest China | Ningxia | Yinchuan | Hong Luan |
| 90 | The First People’s Hospital of Yunnan Province (Kunhua Hospital) | Southwest China | Yunnan | Kunming | Hong Zhang |
| 91 | The Central Hospital of Zhoukou | Central China | Henan | Zhoukou | Hualing Liu |
| 92 | Anyang District Hospital | Central China | Henan | Anyang | Hui Liu |
| 93 | Sichuan Provincial People’s Hospital | Northwest China | Sichuan | Chengdu | Jianhong Tao |
| 94 | Mudanjiang Cardiovascular Disease Hospital | Northeast China | Heilongjiang | Mudanjiang | Jianwen Liu |
| 95 | Yichang Central Hospital | Central China | Hubei | Yichang | Jiawang Ding |
| 96 | Qilu Hospital of Shandong University | Eastern China | Shandong | Jinan | Jifu Li |
| 97 | Affiliated Hospital of Jiangsu University | Eastern China | Jiangsu | Zhenjiang | Jinchuan Yan |
| 98 | The First People’s Hospital of Nanning City | Southern China | Guangxi | Nanning | Jinru Wei |
| 99 | The First Affiliated Hospital of Fujian Medical University | Eastern China | Fujian | Fuzhou | JinziSu |
| 100 | Chengdu Third People’s Hospital | Northwest China | Sichuan | Chengdu | Jiong Tang |
| 101 | Yantaishan hospital | Eastern China | Shandong | Yantai | Juexin Fan |
| 102 | Qingdao Municipal Hospital | Eastern China | Shandong | Qingdao | Jun Guan |
| 103 | Zhongshan Hospital Affiliated to Fudan University | Eastern China | Shanghai | Shanghai | Junbo Ge |
| 104 | Longyan First Hospital | Eastern China | Fujian | Longyan | Kaihong Chen |
| 105 | Affiliated Hospital of Guangdong Medical College | Southern China | Guangdong | Guangzhou | Keng Wu |
| 106 | Jiangxi Provincial People’s Hospital | Eastern China | Jiangxi | Nanchang | Lang Ji |
| 107 | Anhui Provincial Hospital | Eastern China | Anhui | Hefei | Likun Ma |
| 108 | Xiangtan City Central Hospital | Central China | Hunan | Xiangtan | Lilong Tang |
| 109 | The First Hospital of Haerbin City | Northeast China | Heilongjiang | Harbin | Lin Wei |
| 110 | Central Hospital Affiliated to Shenyang Medical College | Northeast China | Liaoning | Shenyang | Man Zhang, Kaiming Chen |
| 111 | The Central Hospital of Wuhan | Central China | Hubei | Wuhan | Manhua Chen |
| 112 | Hangzhou First People’s Hospital | Eastern China | Zhejiang | Hangzhou | Ningfu Wang |
| 113 | The Central Hospital of Xuzhou | Eastern China | Jiangsu | Xuzhou | Peiying Zhang |
| 114 | The Second hospital of Dalian Medical University | Northeast China | Liaoning | Dalian | Peng Qu |
| 115 | The First Affiliated Hospital of Liaoning University of Traditional Chinese Medicine | Northeast China | Liaoning | Shenyang | Ping Hou |
| 116 | Beijing Tsinghua Changgung Hospital | Northern China | Beijing | Beijing | Ping Zhang |
| 117 | Guizhou Provincial People’s Hospital | Southwest China | Guizhou | Guiyang | Qiang Wu |
| 118 | The First Affiliated Hospital of Xiamen University | Eastern China | Fujian | Xiamen | QiangXie |
| 119 | Quanzhou First Hospital | Eastern China | Fujian | Quanzhou | Rong Lin |
| 120 | Wuzhou People’s Hospital | Southern China | Guangxi | Wuzhou | Shaowu Ye |
| 121 | The Central Hospital of Jilin | Northeast China | Jilin | Changchun | Shuangbin Li |
| 122 | Xiangya Hospital Central South University | Central China | Hunan | Changsha | Tianlun Yang |
| 123 | Guangzhou Red Cross Hospital | Southern China | Guangdong | Guangzhou | Tongguo Wu |
| 124 | The First Affiliated Hospital of Guangzhou Medical College | Southern China | Guangdong | Guangzhou | Wei Wang |
| 125 | The First Affiliated Hospital of Wenzhou Medical University | Eastern China | Zhejiang | Wenzhou | Weijian Huang |
| 126 | The Second Affiliated Hospital of Soochow University | Eastern China | Jiangsu | Suzhou | Weiting Xu |
| 127 | Wuhan Asia Heart Hospital | Central China | Hubei | Wuhan | Xi Su |
| 128 | The First Affiliated Hospital of Soochow University | Eastern China | Jiangsu | Suzhou | Xiangjun Yang |
| 129 | Affiliated Hospital of Yan’an University | Northwest China | Shaanxi | Yan’an | Xiaochuan Ma |
| 130 | The First People’s Hospital of Jining | Eastern China | Shandong | Jining | Xiaofei Sun |
| 131 | The Central Hospital of Taiyuan | Northern China | Shanxi | Taiyuan | Xiaoping Chen |
| 132 | West China Hospital of Sichuan University | Northwest China | Sichuan | Chengdu | Xiaoping Chen |
| 133 | The Third Affiliated Hospital of Guangzhou Medical College | Southern China | Guangdong | Guangzhou | Ximing Chen |
| 134 | The First Affiliated Hospital of Wannan Medical College | Eastern China | Anhui | Wuhu | Xingsheng Tang |
| 135 | Tangdu Hospital of The Fourth Military Medical University | Northwest China | Shaanxi | Xi’an | Xue Li |
| 136 | Shanghai East Hospital Affiliated to Tongji University | Eastern China | Shanghai | Shanghai | Xuebo Liu |
| 137 | Xiamen Cardiovascular Disease Hospital | Eastern China | Fujian | Xiamen | Yan Wang |
| 138 | Zhongnan hospital of Wuhan University | Central China | Hubei | Wuhan | Yanggan Wang |
| 139 | Fujian Provincial Hospital | Eastern China | Fujian | Fuzhou | Yansong Guo |
| 140 | The First Affiliated hospital of Dalian Medical University | Northeast China | Liaoning | Dalian | Yanzong Yang |
| 141 | The First People’s Hospital of Changde | Central China | Hunan | Changde | Yi Huang |
| 142 | The First Affiliated Hospital of China Medical University | Northeast China | Liaoning | Shenyang | Yingxian Sun |
| 143 | The Fourth Affiliated Hospital of China Medical University | Northeast China | Liaoning | Shenyang | YuanzheJin |
| 144 | Cangzhou Central Hospital | Northern China | Hebei | Cangzhou | Zesheng Xu |
| 145 | The Central Hospital of Shaoyang | Central China | Hunan | Shaoyang | Zewei Ouyang |
| 146 | The People’s Hospital of Liaoning Province | Northeast China | Liaoning | Shenyang | Zhanquan Li |
| 147 | The First Affiliated Hospital of Jiamusi University | Northeast China | Heilongjiang | Jiamusi | Zhaofa He |
| 148 | Tangshan Gongren Hospital | Northern China | Hebei | Tangshan | Zheng Ji |
| 149 | Huaibei Miners General Hospital | Eastern China | Anhui | Huaibei | ZhenqiSu |
| 150 | Linyi People’s Hospital | Eastern China | Shandong | Linyi | ZhihongOu |
| 151 | Chongqing Hechuan District People’s Hospital | Southwest China | Chongqing | Chongqing | Xin Tang |
| 152 | Yuzhou City Central Hospital | Central China | Henan | Xuchang | QinfengSu |
| 153 | Jianshui County People’s Hospital | Southwest China | Yunnan | Honghe | Weiqing Fan |
| 154 | Dunhua City Hospital | Northeast China | Jilin | Dunhua | Fanju Meng |
| 155 | Shenyang City Electricity Central Hospital | Northeast China | Liaoning | Shenyang | Jing Xu |
| 156 | Shanghai Jingan District Shibei Hospital | Eastern China | Shanghai | Shanghai | Bin Wang |
| 157 | Beijing Fangshan District First Hospital | Northern China | Beijing | Beijing | Xuemei Peng |
| 158 | Hebei Daming County People’s Hospital | Northern China | Hebei | Handan | Haiping Guo |
| 159 | Jiangsu Binhai County People’s Hospital | Eastern China | Jiangsu | Yancheng | Yonglin Zhang |
| 160 | The First People’s Hospital of Longquanyi District | Southwest China | Sichuan | Chengdu | Wei Tuo |
| 161 | Guangxi Hengxian County People’s Hospital | Southern China | Guangxi | Nanning | Xianan Zhang |
| 162 | Hunan Changsha County First People’s Hospital | Central China | Hunan | Changsha | Siding Wang |
| 163 | People’s Hospital of Wugang | Central China | Hunan | Shaoyang | JiaoMei Yang |
| 164 | Longhui County People’s Hospital | Central China | Hunan | Shaoyang | Xiaojun Wang |
| 165 | Heilongjiang Fujin City Central Hospital | Northeast China | Heilongjiang | Jiamusi | Jiyan Yin |
| 166 | Dalian Fourth People’s Hospital | Northeast China | Liaoning | Dalian | Huifang Zhang |
| 167 | General Hospital of Guangzhou Military Command | Southern China | Guangdong | Guangzhou | Yanlie Zheng |
| 168 | The First People’s Hospital of Horqin District, Tongliao City | Northern China | Inner Mongolia | Tongliao | Junping Fang |
| 169 | Guiyang Sixth People’s Hospital | Southwest China | Guizhou | Guiyang | Kalan Luo |
| 170 | Geological Mining Hospital of Hunan Province | Central China | Hunan | Changsha | Naiyi Liang |
| 171 | Zhangzhou Municipal Hospital of Fujian Province | Eastern China | Fujian | Zhangzhou | Changyong Liu |
| 172 | Jining City Yanzhou District People’s Hospital | Eastern China | Shandong | Jining | Jian Yang |
| 173 | The People’s Hospital Feixian | Eastern China | Shandong | Linyi | Honghua Deng |
| 174 | Tangshan City Fengrun District People’s Hospital | Northern China | Hebei | Tangshan | Lin Wang |
| 175 | Qian’an People’s Hospital | Northern China | Hebei | Tangshan | Yuheng Yang |
| 176 | Yuzhong County People’s Hospital | Northwest China | Gansu | Lanzhou | Xiaowei Peng |
| 177 | Baiyin Cite Center Hospital | Northwest China | Gansu | Baiyin | Fang Zhao |
| 178 | Mingguang People’s Hospital | Eastern China | Anhui | Chuzhou | Yong Li |
| 179 | Xihua County People’s Hospital | Central China | Henan | Zhoukou | Chuntong Wang |
| 180 | Zhalantun People’s Hospital | Northern China | Inner Mongolia | Hulunbeier | Yuhua Zhu |
| 181 | Fengrun District Second People’s Hospital | Northern China | Hebei | Tangshan | Jingshan Zhao |
| 182 | Zhangping City Hospital | Eastern China | Fujian | Zhangpin | Jinxing Yi |
| 183 | Fuqing Cite Hospital | Eastern China | Fujian | Fuqing | Ping Chen |
| 184 | The Eight Affiliated Hospital, Sun Yat-sen University | Southern China | Guangdong | Guangzhou | Nan Jia |
| 185 | The Second Affiliated Hospital of Qiqihar Medical University | Northeast China | Heilongjiang | Qiqihar | Yanli Wang |
| 186 | Wuhan University of Science and Technology Hospital | Central China | Hubei | Wuhan | Jing Hu |
| 187 | Baotou City Center Hospital | Northern China | Inner Mongolia | Baotou | Ruiping Zhao |
| 188 | Shanghai Jiading District Center Hospital | Eastern China | Shanghai | Shanghai | Xia Chen |
| 189 | Datong City Second People’s Hospital | Northern China | Shanxi | Datong | Xiaoqin Zhang |
| 190 | Binyang People’s Hospital | Southern China | Guangxi | Binyang | Fudong Gan |
| 191 | Deqing People’s Hospital | Eastern China | Zhejiang | Deqing | Fangfang Huang |
| 192 | Xinmi people’s hospital | Central China | Henan | Xinmi | Xiaolei Li |
| 193 | Dongguan Changping hospital | Southern China | Guangdong | Dongguan | Haiyun Lin |
| 194 | Gongyi people’s hospital | Central China | Henan | Gongyi | Tianmin Du |
| 195 | Ye County people’s hospital | Central China | Henan | Yexian | Jie Yang |
| 196 | The second people’s hospital of Mengcheng | Eastern China | Anhui | Mengcheng | Pengfei Zhang |
| 197 | Nanpi People’s Hospital | Northern China | Hebei | Nanpi | Hui Dong |
| 198 | Shimen People’s Hospital | Central China | Hunan | Shimeng | Chuanliang Liang |
| 199 | Tieli People’s Hospital | Northeast China | Heilongjiang | Tieli | YanboNiu |
| 200 | Sihui People’s Hospital | Southern China | Guangdong | Sihui | Yuehua Huang |
| 201 | Chest Hospital of Xinjiang Uygur Autonomous Region | Northwest China | Xinjiang | Urumchi | Dongsheng Chai |
| 202 | Beian First People’s Hospital | Northeast China | Heilongjiang | Bei’an | Dongyan Li |
| 203 | Zunhua People’s Hospital | Northern China | Hebei | Zunhua | Xiaoli Yang |
| 204 | Lujiang People’s Hospital | Eastern China | Anhui | Lujiang | Qichun Wang |
| 205 | Qinyang People’s Hospital | Central China | Henan | Qinyang | Xiaowen Ma |
| 206 | Longmen People’s Hospital | Southern China | Guangdong | Longmen | Yingchao Luo |
| 207 | QuyangRenji Hospital | Northern China | Hebei | Quyang | Congliang Zhang |
| 208 | Nenjiang People’s Hospital | Northeast China | Heilongjiang | Nenjiang | Shuhua Zhang |
| 209 | Longjiang First People’s Hospital | Northeast China | Heilongjiang | Longjiang | Yuhuan Shi |
| 210 | Li County Hospital of Traditional Chinese Medicine | Central China | Hunan | Changde | Songbai Li |
| 211 | Luan County People’s Hospital | Northern China | Hebei | Luanxian | Guo Li |
| 212 | Yulong Hospital | Southwest China | Yunnan | Yulong | Zeyuan He |
| 213 | Huining People’s Hospital | Northwest China | Gansu | Huining | Jiabin Xi |
| 214 | Yuncheng Hospital | Eastern China | Shandong | Yuncheng | JinglanDiao |
| 215 | Hepu People’s Hospital | Southern China | Guangxi | Hepu | Meisheng Lai |
| 216 | Duzishan Petrochemical Hospital | Northwest China | Xinjiang | Dushanzi | Shuqiu Qu |
| 217 | Guiding People’s Hospital | Southwest China | Guizhou | Guiding | Guoduo Chen |
| 218 | People’s Hospital of Rongchang District | Southwest China | Chongqing | Chongqing | Jie Chen |
| 219 | Ningbo First Hospital | Eastern China | Zhejiang | Ningbo | Huimin Chu |
| 220 | Ledong Second People’s Hospital | Southern China | Hainan | Ledong | Xiufeng Chen |
| 221 | Guang’an People’s Hospital | Southwest China | Sichuan | Guang’an | Tian Tuo |
| 222 | Linfen People’s Hospital | Northern China | Shanxi | Linfen | Junping Deng |
| 223 | People’s Hospital of Bozhou District | Southwest China | Guizhou | Zunyi | Shengyong Chen |
| 224 | Dianjiang People’s Hospital | Southwest China | Chongqing | Dianjiang | Yang Yu |
| 225 | First Affiliated Hospital of Harbin Medical University. | Northeast China | Heilongjiang | Harbin | Yue Li |
| 226 | Yiliang Hospital | Southwest China | Yunnan | Yiliang | Liqiong Yang |
| 227 | HaidongPing’an District Hospital of Traditional Chinese Medicine | Northwest China | Qinghai | Haidong | Guoqin Xin |
| 228 | Ningjin People’s Hospital | Eastern China | Shandong | Ningjin | Tao Zhang |
| 229 | Yutian Hospital | Northern China | Hebei | Yutian | Xiaoyun Feng |
| 230 | Yanting People’s Hospital | Southwest China | Sichuan | Yanting | Mingcheng Bai |
| 231 | The Fourth Affiliated Hospital Zhejiang University School of Medicine | Eastern China | Zhejiang | Yiwu | Shudong Xia |
| 232 | Wuxi Xishan People’s Hospital | Eastern China | Jiangsu | Wuxi | Xudong Li |
| 233 | Dongfeng Hospital | Northeast China | Jilin | Dongfeng | Wei Liu |
| 234 | Zhijin People’s Hospital | Southwest China | Guizhou | Zhijin | Zhongshan Wang |
| 235 | Huaiyang People’s Hospital | Central China | Henan | Huaiyang | Li Wei |
| 236 | Suizhou Central Hospital | Central China | Hubei | Suizhou | Fengwei Li |
| 237 | Tonglu First People’s Hospital | Eastern China | Zhejiang | Tonglu | Xiaolan Li |
| 238 | Xiantao First People’s Hospital | Central China | Hubei | Xiantao | Dongmei Zhu |
| 239 | Honghu People’s Hospital | Central China | Hubei | Honghu | Hong Liu |
| 240 | Xinjin County Hospital of Traditional Chinese Medicine | Northwest China | Sichuan | Xinjin | YingbiSu |
